# Requests and apologies in two languages among bilingual speakers: A comparison of heritage English speakers and English- and Hebrew-dominant bilinguals

**DOI:** 10.3389/fpsyg.2022.1017715

**Published:** 2022-12-22

**Authors:** Sagit Bar On, Natalia Meir

**Affiliations:** ^1^Department of English Literature and Linguistics, Bar Ilan University, Ramat Gan, Israel; ^2^The Gonda Multidisciplinary Brain Research Center, Bar-Ilan University, Ramat Gan, Israel

**Keywords:** heritage language, L2, English, Hebrew, pragmatics

## Abstract

**Introduction:**

Linguistic research over the last two decades has uncovered a significant number of properties that identify heritage language (HL) speakers as a particular phenomenon within bilingualism. However, despite the rapid development of HL research, the sphere of HL speech act pragmatics is still in its infancy.

**Methodology:**

The current study sought to cover part of this gap by investigating both languages of HL adult speakers for whom English is their HL and Hebrew is their dominant societal language (SL; *n* = 20, hereafter HS) in comparison with two other groups: Hebrew-dominant speakers who were born to Hebrew-speaking families and raised in Israel, and thus English is the language of their schooling (*n* = 20, hereafter HEB-D), and English-dominant speakers who were born to English-speaking families and immigrated to Israel from an English-speaking country after the age of 18, and thus Hebrew is their L2 (*n* = 20, hereafter ENG-D). The discourse-pragmatic tasks in English and in Hebrew consisted of the same 36 scenarios eliciting requests and apologies in each language. Each request was followed by an apology that is related.

**Results:**

The results indicated that Hebrew-dominant speakers and English-dominant speakers, i.e., HEB-D and ENG-D, had different strategies for the realization of requests and apologies which they systematically transferred from their dominant language into their weaker one, confirming the cross-cultural and cross-linguistic differences between request and apology realizations in English and in Hebrew. However, the picture was more complex for the HL speakers in their HL-English and SL-Hebrew as in some cases their strategies paired up with the ENG-D in English, and with the HEB-D in Hebrew, while in other cases they developed a unique and hybrid linguistic style reflecting the strategies of both languages.

**Discussion:**

The investigation of both languages of HL speakers enabled us to compare features of the two linguistic systems and make conclusions about their nature.

## Introduction

Despite the rapid development of Heritage Language (HL) research, the sphere of speech act pragmatics has remained uncharted territory. The current study is devised to investigate pragmatic abilities of HL speakers of English who have acquired their HL-English in contact with Hebrew as the dominant SL. The pragmatic abilities of the HL-English speakers are investigated *via* comparing their request and apology realizations in English to English-dominant speakers (L1-English, L2-Hebrew), and in Hebrew to Hebrew-dominant speakers (L1-Hebrew, L2-English). The investigation of both languages, HL-English and SL-Hebrew, is expected to provide a more comprehensive picture of the pragmatic abilities of this unique bilingual group. It is important to note that the HL-speakers in this study might be more balanced bilinguals than prototypical HL speakers due to the fact that English is a mandatory subject in the Israeli national education system.

HL speakers are bilinguals who are exposed to their HL from birth *via* naturalistic input at home, yet the HL is not the dominant language of the larger surrounding society (Rothman, 2009; Montrul, 2018; Polinsky, 2018). Thus, HL speakers use and acquire their HL in a socio-linguistically, socio-culturally, and socio-politically complex situation (Xiao-Desai, 2019). HL speakers are considered to be asymmetrical bilinguals since they learned their HL as a first language in childhood at home, but, as adults, they become dominant in the majority societal language (SL) spoken by the community (Elabbas et al., 2013). There are two main factors which are reported to influence the linguistic systems of HL speakers: HL input characteristics and cross-linguistic influence (Montrul, 2016; Polinsky, 2018). Input relates to the timing, amount, and content of the exposure to the languages, while cross-linguistic influence is concerned with the effect that one language has on the other.

Numerous studies have discussed connections between language, culture, and identity (Wallace, 2004; Berard, 2005; Achugar, 2006). HL speakers belong simultaneously to two socio-linguistic and socio-cultural communities. The core identity of HL speakers involves the process of constant negotiation and self-positioning within a bilingual and bicultural environment (Val and Vinogradova, 2010). Rothman (2009) claims that HL speakers feel strong pressure to assimilate to the mainstream culture, and therefore, gradually begin to use their SL more and more at home. As a result, patterns of the HL gradually change and become modified in quality. Thus, by the time HL children reach adolescence and young adulthood, their HL might resemble, in some aspects, an L2 learned in adulthood as opposed to L1 acquired in childhood.

Most studies in interlanguage pragmatics investigate influences from L1 to L2, and do not consider a bi-directional relationship between the two languages (Cenoz, 2003). However, Blum-Kulka (1990) and Blum-Kulka and Sheffer (1993) have found that the realization patterns of requests produced in English (L1) and in Hebrew (L2) by American immigrants to Israel who were fully competent in the two languages differed significantly from both the Israeli and the American patterns. Their requests presented features that can be situated in between American and Israeli requests. Blum-Kulka (1991) proposes the ‘Intercultural Style Hypothesis’ to define the development of an intercultural pattern that reflects bi-directional interactions between the languages. Cenoz (2003) found that native Spanish speakers who achieved a high level of proficiency in English developed an intercultural pattern that was reflected both in the similarity between their requests production in Spanish and in English and in the differences between these requests and those formulated by other native speakers of Spanish. These findings support the ‘Intercultural Style Hypothesis’ and show that the interaction between the languages of a proficient multilingual speaker is bi-directional. Research investigating immigrants in other countries is also compatible with this hypothesis. For example, Su (2010) investigated Chinese learners of English requesting behavior in both their L1 and L2 and found a bi-directional transfer at a pragmatic level. Kecskes and Papp (2000) also examined the influence of foreign language learning on the development of mother tongue skills from a cognitive-pragmatic perspective and found evidence that foreign language acquisition can influence different areas of L1 when exposure to the foreign language is intensive. However, as Kasper and Blum-Kulka (1993) point out, more research is needed in the field of intercultural pragmatics.

Cross-linguistic transfer and shrinkage of grammatical structures have been related to economy principles under which the mind favors the least amount of effort toward a cognitive task. Therefore, humans organize knowledge in their brain by dividing it into ‘classes’ and/or match patterns to reduce cognitive load. This means that HL speakers might resort to simpler structures that overlap in both languages (Rothman, 2015; Polinsky and Scontras, 2020) and/or develop a unique and hybrid intercultural linguistic style reflecting both their SL and their HL (Pinto and Raschio, 2007; Xiao-Desai, 2019) in order to reduce the cognitive load associated with being bilinguals, e.g., inhibition costs.

Most of the previous HL research has addressed morpho-syntactic competence of HL speakers (for an extensive overview see Montrul, 2018; Polinsky, 2018), whereas the available research on pragmatic abilities of HL speakers is still very limited and covers mainly Spanish and Russian as HLs (see Pinto and Raschio, 2007; Dubinina, 2011; Dubinina and Malamud, 2017; Xiao-Desai, 2019).

### Request strategies: Differences between English and Hebrew speakers

Requests are by definition face-threatening acts. The notion of ‘face’ can be divided into two concepts: positive face and negative face. Positive face is the need to be accepted, liked by others, treated as a member of the group, and know that his/her wants are shared by others. Negative face is the need to be independent to have freedom of action, and not to be imposed upon by others. By making a request, the speaker impinges on the hearer’s right to freedom of action and from imposition. Therefore, requests are considered to be face-threating acts in which the negative face of the hearer is threatened, and when ‘face’ is threatened speakers typically act to mitigate that threat by doing ‘facework’ (Brown and Levinson, 1987).

Requests have been divided in the literature into three main segments: Alerters, head acts, and supportive moves. Alerters are opening elements that precede the actual request and are primarily used to get the hearer’s attention. They are optional to the realization of the request and can come in the form of address terms or attention-getters (Blum-Kulka and Olshtain, 1984). Head acts are the core part of the request sequence which realize a request independently of other elements. The head acts are the actual requests and serve as an integral part of any request. A request might contain more than one head act (Bella, 2012). Supportive moves are adjuncts to the head acts used to modify the impact or force of requests. However, there are cross-linguistic and cross-cultural differences in request realizations. For example, one way in which the speaker can soften the imposition is by choosing an indirect strategy over a direct one (Blum-Kulka and Olshtain, 1984).

In both English and Hebrew, head acts of requests can be grammatically realized with imperatives, interrogatives, and declaratives (Blum-Kulka and Olshtain, 1984; Curl and Drew, 2008). However, even though in English direct imperatives are usually defined as appropriate constructions for commands or instructions, they are less appropriate for making requests (Márquez-Reiter, 2000). Searle (1975) claims that the “ordinary conversational requirements of politeness normally make it awkward to issue flat imperatives, and we therefore seek to find indirect means to our illocutionary needs.” Blum-Kulka and Olshtain (1984) explain that even though imperatives in Hebrew are considered appropriate for requesting, they are the most direct and explicit level among the syntactic structures available for making requests, and therefore the least polite constructions. Olshtain and Blum-Kulka (1985) noted that in an acceptability judgment test which they developed, English speakers who were learners of L2 Hebrew (and living in Israel for a period of less than 2 years) were reluctant to accept the direct request strategy found in Hebrew native speech. For example, the L2 learners were inclined to reject the Hebrew equivalent of the declarative ‘I hope you can take me back to town’ when asking for a ride, whereas native Hebrew speakers had no problem accepting it. The learners have responded in accordance with the politeness norms for requesting in their L1 (the most common structure to realize a request in English is to use an interrogative structure combined with a modal verb).

Modals can be used with both declarative and interrogative sentences (Walters, 1979). However, since linguistic expressions of modality convey speakers’ claims about the permission, ability, probability, possibility, etc. of beliefs and actions, and therefore have a notion of indirectness to them (Turnbull and Saxton, 1997) there are likely to be more frequent in an English speech than in Hebrew.

Although *‘please*’ (and its equivalents) is a universal mitigating device (Ogiermann, 2009; Murphy and De Felice, 2018; Webman-Shafran, 2019), there are cross-cultural and cross-linguistic differences with respect to its usage. Dubinina and Malamud (2017) suggest that the marker *‘please’* in English can be used only in conventional requests, both direct (e.g., *‘Do this exercise, please!’*) and indirect (*‘Could you open the door please?’*). However, it is not allowed in utterances that do not have the form conventionally used for requests, even when their form and propositional content are similar to conventional requests, and even if these are ultimately interpreted as requests [e.g., ‘*Are you able to open the door (#please)?*’]. Nonetheless, according to House (1989) this politeness marker is most appropriate in mitigating ‘standard situations’ where the request making and the fulfillment of it are self-evident and the function of the request is clear. However, when speakers prefer to disguise the function of the request in the form of a question, they tend to leave out the ‘please’ marker in order to allow the addressee to respond to the propositional content of the utterance and not reveal its conventional function.

To sum up, in English, making a request short and straightforward is considered impolite and face threatening. Therefore, in order to save ‘face’ English speakers prefer to use a longer and indirect version of a request by applying interrogatives and modals. However, mitigating a request by using an interrogative structure (i.e., a question) with a modal makes the use of ‘*please’* somewhat redundant. Hebrew speakers, on the other hand, prefer a more straightforward strategy in the form of a declarative (statement). Yet, in order to mitigate ‘face threatening’, and as the intent of requesting is already visible, they apply the marker ‘*bevakasha*’ (*‘please’*).

### Apology strategies: Differences between English and Hebrew speakers

Apologies are also considered face-threatening acts (Brown and Levinson, 1987), however, contrary to requests which are ‘pre-event acts’, apologies are ‘post-event acts’. In other words, while requests are made to cause an event or to change one, apologies signal an event of a social norm violation that has already taken place which the speaker holds himself/herself at least partially accountable for (Leech, 1980; Blum-Kulka and Olshtain, 1984). Therefore, as opposed to requests, apologies are face-threating acts in which the negative face of the speaker is threatened.

The speech act of apology is universal, yet its strategies and linguistic variations differ cross-culturally to a great extent (Israa, 2017). Blum-Kulka and Olshtain (1984) explain that a strategy for the act of apologizing can come in one of two basic forms (or a combination of both): (i) Direct realization of an apology *via* an explicit illocutionary force-indicating device (hereafter IFID), i.e., *via* performative expressions such as ‘sorry’, ‘apologize’, ‘forgive’, or ‘pardon’, and (ii) Indirect realization of an apology *via* the use of an utterance which contains reference to one or more elements from a closed set of four specified propositions, i.e., explaining the cause, acknowledging responsibility, offering repair, and promising forbearance.

Cohen and Olshtain (1981) investigated apologies of Hebrew speakers that learned English as an L2, and discovered that the L2-English learners with L1-Hebrew did not seem to be familiar with the accepted formulas needed for the apologies in English. To be more specific, Hebrew speakers learners of English were less likely to accept responsibility for an offense or to make an offer for repair than native English speakers. This is in line with Mills and Grainger’s (2016) claim that in Hebrew there is a tendency for directness to be evaluated positively as part of the Israeli cultural style, in contrast to Anglo-European norms which are indirect and often characterized as overly mannered. For instance, they found that Hebrew speakers were comfortable with direct statements in a way that British English speakers were not as a result of conceptual ideology about directness which Hebrew speakers perceive as signaling honesty and friendliness. Ellis and Maoz (2002) add that Hebrew speech is characterized as direct and “to the point,” and is used both within the culture and during intercultural communication. This ‘dugri’ (straightforward) style of the Israeli culture enables Hebrew speakers to use an explicitness about intentions that in other cultures could be considered offensive (Katriel, 1986). Thus, bearing in mind the directness that characterizes Hebrew, while also taking into consideration Cohen and Olshtain’s (1981) findings, it should not be surprising that Hebrew speakers prefer to use the direct strategy of expressing an explicit IFID, while English speakers prefer to use the indirect strategy of choosing one or more of the other indirect propositions.

In addition to these main strategies, which make up the speech act of the apology itself, there are ways in which the speaker can modify the apology, e.g., by performing it with different levels of intensity using intensification terms such as ‘so’, ‘very’, ‘really’, ‘terribly’, ‘extremely’, ‘totally’, ‘deeply’, and ‘highly’. Previous studies show that there are differences across speakers of different languages in the use of intensification. For example, Cohen et al. (1986) found that native Hebrew speakers who were learners of L2-English intensified their apologies significantly more than native English speakers, even though this extra intensity on the part of the Hebrew speakers was not necessarily warranted given the generally low or moderate severity of the offense. The same trend was found in Olshtain (1983) where English and Russian learners of Hebrew did not intensify apologies in a target-like manner.

To sum up, English speakers rely more on indirect strategies compared to Hebrew speakers. In regard to apologies this means that English speakers prefer the less direct strategy of propositions, while Hebrew speakers prefer the direct strategy of IFIDs. Furthermore, the few studies that have investigated the usage of adverbial intensifiers in English and in Hebrew show that Hebrew speakers tend to intensify their apologies more than English speakers.

### The current study

This study aimed at providing a comprehensive overview of HL-English speakers’ linguistic behavior by comparing their realization patterns of two speech acts, requests and apologies, in both of their languages, i.e., English and Hebrew, to two groups with varying level of dominance: Hebrew-dominant speakers and English-dominant speakers. Research comparing both languages of HL speakers is limited. To the best of our knowledge, only three studies investigated both languages of HL speakers: Scontras et al. (2017) investigated the interpretation of ‘every’ in HL-Mandarin and SL-English; Kupisch et al. (2014) investigated accentedness in two different HL-SL dyads, German-French and German-Italian; and Stangen et al. (2015) investigated accentedness in HL-Turkish and SL-German. A comparison of requests and apologies in the two languages of bilingual subjects is expected to shed light on the mechanisms of cross-linguistic and cross-cultural realization of politeness under diminished input (quantitatively and qualitatively), and adds to the growing body of literature concerning politeness and speech acts in English and in Hebrew. Research on HL-English is rather limited (Polinsky, 2018), as English is mainly studied as the L2. Yet Israel offers a rare opportunity to investigate HL-English. The uniqueness of English as the HL is that it is the *de facto* international language of the modern world, on the one hand, but it also patterns with other HLs as it undergoes divergence in contact situations where it is a minority language (Meir et al., 2021).

The study aimed to answer to what extent request and apology strategies produced in English and in Hebrew by HL-English speakers differ from or resemble the ones produced by the English-dominant speakers (i.e., speakers who were born to English-speaking families and raised in an English-speaking country) and/or by the Hebrew-dominant speakers (i.e., speakers who were born to Hebrew-speaking families and raised in Israel).

For this objective, three hypotheses were formulated: (H1) HL speakers will show deviation in the production of realization patterns and carry over pragmatic and socio-linguistic behavior from their HL-English into their SL–Hebrew, (H2) HL speakers will show deviation in the production of realization patterns and carry over pragmatic and socio-linguistic behavior from their SL-Hebrew into their HL–English, and (H3) HL speakers will show a hybrid pragmatic competence, i.e., they will show evidence of developing new conventions for the production of realization patterns, which differ from the corresponding conventions of the other two groups.

## Methodology

### Participants

The study comprised three groups of adult bilingual speakers between the ages of 23–30: (i) HL-English bilinguals who were dominant in Hebrew, born to English-speaking families but raised in Israel from birth or arrived in Israel before the age of five (HS); (ii) Hebrew-dominant bilinguals, born to Hebrew-speaking families and raised in Israel, and thus English is the language of their schooling (HEB-D); and (iii) English-dominant bilinguals, born to English-speaking families and immigrated to Israel from an English-speaking country after the age of 18 (ENG-D). All groups were tested in both English and Hebrew. All three groups were balanced in regard to the number of participants and the gender, i.e., ten females and ten males were tested in each group (a total of sixty participants in the study). All participants were of medium to high socio-economic status as suggested by the level of their education. The minimum age of 23 was chosen in order to make sure that the participants in the ENG-D group would have sufficient amount of proficiency in Hebrew to complete the tasks.

All participants were asked to fill in a self-report background questionnaire eliciting the participants’ demographic information such as age, gender, level of education, occupation, birthplace, place of residence, etc., as well as language-related information such as age of onset (the age at which each language was acquired or learned), proficiency in all four domains of language (reading, writing, comprehending, and speaking), frequency of usage, and non-native accent ratings. The participants’ demographic data are shown in Table 1, and the self-evaluated language information is shown in Table 2. Tables 1 and 2 present data on the participants per group, per language, and statistics for group differences.

**Table 1:** Participants’ demographic data [Mean (SD)].

	HS (*N* = 20)	HEB-D (*N* = 20)	ENG-D (*N* = 20)	Group differences	Tukey HSD *Post hoc* analysis for multiple comparisons
Age (Years)	25.4 (2.4)	25.7 (2.4)	26.3 (2.8)	*F*(2,57) = 0.545, *p* = 0.583	n/a
Gender	F = 10 M = 10	F = 10 M = 10	*F* = 10 M = 10	*χ2* = 0, *p* = 1.00	n/a
Education (Years)	15.3 (1.8)	15.5(1.8)	14.9 (0.9)	*F*(2,57) = 0.725, *p* = 0.489	n/a
Immigration to Israel (Age)	1.45 (2)	0 (0)	18.3 (0.6)	*F*(2,57) = 1,297, *p* = <0.001	HEB-D < HS < ENG-D
Age of Onset of English	0 (0)	9 (0)	0 (0)	*F*(2,57) = 180.7, *p* < 0.001	(ENG-D=HS) < HEB-D
Age of Onset of Hebrew	0.8 (1.6)	0 (0)	14.4 (4.2)	*F*(2,57) = 180.7, *p* < 0.001	(HEB-D=HS) < ENG-D

**Table 2:** Participants’ subjective ratings of proficiency [Mean (SD)].

	HS (*N* = 20)	HEB-D (*N* = 20)	ENG-D (*N* = 20)	Group differences	Tukey HSD *Post hoc* analysis for multiple comparisons
English proficiency in reading (1–7)	6.4 (0.5)	4.2 (0.6)	7 (0)	*F*(2,57) = 163.8, *p* < 0.001	HEB-D < HS < ENG-D
English proficiency in writing (1–7)	6.3 (0.5)	3.8 (0.7)	7 (0)	*F*(2,57) = 186.2, *p* < 0.001	HEB-D < HS < ENG-D
English proficiency in comprehending (1–7)	7 (0)	5.5 (0.4)	7 (0)	*F*(2,57) = 161.4, *p* < 0.001	HEB-D < (ENG-D=HS)
English proficiency in speaking (1–7)	6.6 (0.4)	4.5 (0.4)	7 (0)	*F*(2,57) = 202, *p* < 0.001	HEB-D < HS < ENG-D
Total English proficiency (4–28)	26.3 (1.4)	18.2 (0)	28 (0)	*F*(2,57) = 249.3, *p* < 0.001	HEB-D < HS < ENG-D
Hebrew proficiency in reading (1–7)	7 (0)	7 (0)	4.2 (0.8)	*F*(2,57) = 209, *p* < 0.001	ENG-D < (HEB-D=HS)
Hebrew proficiency in writing (1–7)	7 (0)	7 (0)	4 (0.8)	*F*(2,57) = 255.4, *p* < 0.001	ENG-D < (HEB-D=HS)
Hebrew proficiency in comprehending (1–7)	7 (0)	7 (0)	6.1 (0.5)	*F*(2,57) = 53.07, *p* < 0.001	ENG-D < (HEB-D=HS)
Hebrew proficiency in speaking (1–7)	7 (0)	7 (0)	5.3 (0.8)	*F*(2,57) = 77.34, *p* < 0.001	ENG-D < (HEB-D=HS)
Total Hebrew proficiency (4–28)	28 (0)	28 (0)	19.7 (2.7)	*F*(2,57) = 169.8, *p* < 0.001	ENG-D < (HEB-D=HS)
Degree of accentedness in English (1–7)	1.3 (0.4)	6.2 (0.6)	1 (0)	*F*(2,57) = 829.2, *p* < 0.001	(HS = ENG-D) < HEB-D
Degree of accentedness in Hebrew (1–7)	1 (0)	1(0)	5 (0.9)	*F*(2,57) = 328.9, *p* < 0.001	(HS=HEB-D) < ENG-D
Current exposure to English (0–100)	29 (7.6)	0 (0)	69 (7.6)	*F*(2,57) = 579.8, *p* < 0.001	HEB-D < HS < ENG-D
Current exposure to Hebrew (0–100)	71 (7.6)	100 (0)	31 (7.6)	*F*(2,57) = 579.8, *p* < 0.001	ENG-D < HS < HEB-D

In order to obtain a more direct measure of the participants’ language proficiency in English and in Hebrew, the vocabulary size in both languages was tested using the Multilingual Naming Test (hereafter MINT; Gollan et al., 2012). The MINT has been validated as an objective proficiency measure for bilingual speakers who speak any combination of English, Spanish, Mandarin, and Hebrew (Tomoschuk et al., 2019). The task included the same 68 black and white picture stimuli. In this task, the participants were asked to say out loud the name of the object they saw in the picture, once in English and once in Hebrew. Each response was coded as correct or incorrect. In line with the profile presented by the participants *via* the questionnaire, the results of the MINT task showed that the HS group paired up with the HEB-D group with respect to their vocabulary size in Hebrew, yet they showed lower proficiency than the ENG-D group in English (even though much higher than HEB-D). Table 3 presents data of the MINT scores of the participants per group, per language, and group comparisons using one-way ANOVAs.

**Table 3:** MINT task performance per group per language (Mean (SD)).

	HS (*N* = 20)	HEB-D (*N* = 20)	ENG-D (*N* = 20)	Group differences	Tukey HSD *Post hoc* analysis for multiple comparisons
MINT-English	57.4 (3.5)	38.6 (10.3)	64.9 (2.3)	F(2,57) = 82.93, *p* < 0.001	HEB-D > HS > ENG-D
MINT-Hebrew	58.4 (2.8)	61.4 (2.7)	40.3 (11.6)	F(2,57) = 49.05, *p* < 0.001	ENG-D > (HS=HEB-D)

### The pragmatic task

A discourse-pragmatic task was designed for this study to elicit requests and apologies. The parallel tasks in English and in Hebrew consisted of 18 scenarios eliciting requests and 18 scenarios eliciting apologies in each language (a total of 36 scenarios in each language). The scenarios were arranged in a random order with pictures showing whether the scenario addressed a female or a male. Each request was followed by an apology that is related (for similar procedure see Márquez-Reiter, 2000). In this task, the participants were asked to say out loud what they would have said if they had been one of the participants in the actual situation while being as spontaneous as possible. In order to take into account variations of the participants’ English and Hebrew literacy skills and to ensure that reading proficiency would not affect the results, the scenarios were read aloud by the experimenter (for similar procedure see Walters, 1979, 1980, 1981; Rintell, 2009 and Dubinina and Malamud, 2017). Examples of the scenarios from the task are presented in Table 4, and the entire list of stimuli is presented in Appendix A. The task manipulated ‘Social Status’ (i.e., the relative level of respect, honor, and deference) and ‘Social Distance’ (i.e., the level of familiarity between the participants), and controlled for gender.

**Table 4:** Examples of scenarios.

The speech act	The preamble
Request 1	You are a student. You conducted research for a seminar paper, but you do not know how to do the statistics. You know that your elderly neighbor is very good at it, and you want to ask him for an hour of his time to help you. You see him in his garden. What do you say to him?
Apology 1	You are a student. You asked your elderly neighbor to help you with statistics for an hour. However, you forgot to show up on time and you are an hour late. What do you say to him?
Request 2	You are on a bus with a child. There are plenty of seats on the bus but there are not any for two people together. You want to ask a passenger who is sitting on her own to change seats with you so that you can sit next to the child. What do you say to her?
Apology 2	A passenger has agreed to change seats with you so that you can be next to a child on the bus. While changing seats you accidentally step on her foot. What do you say to her?

### Coding schemata

The coding schemata adopted in the current study were developed based on several former studies (Cohen and Olshtain, 1981; Blum-Kulka and Olshtain, 1984; Dubinina and Malamud, 2017; Israa, 2017).

There are syntactic, lexical, and structural variations in the production of requests and apologies across different languages. In the current study, the participants’ requests were analyzed for the presence of alerters, head acts, and supportive moves. Furthermore, we also noted the syntactic structure of the head act/s (interrogative/imperative/declarative/mixed-when a request contained two head acts with different syntactic structures), the use of modals, and the use of ‘*please*’/‘*bevakasha*’. The participants’ apologies were analyzed for expressions of apology, number of propositions added (i.e., offering explanation, taking responsibility, offering repair or compensation, and promising forbearance), and the use of adverbial intensifiers.

### Procedure

The participants in the study were recruited by word of mouth through personal social networks. Prior to the data collection, all participants who agreed to volunteer were given a recruitment letter (each in his/her dominant language) explaining the general aim of the study without revealing its specific aim. Upon agreeing to take part in this research, each participant was allocated a personal participant’s code and was asked to fill in a self-report Google Form questionnaire in his/her dominant language using that code for identification. In order to obtain a broad-based sample from a variety of geographical locations some of the participants were tested *via* Zoom. Each participant was tested individually in both English and Hebrew. Testing in each group had been counterbalanced, i.e., ten participants completed the tasks in English followed by Hebrew, and ten participants completed the tasks in Hebrew followed by English. The elicitation tasks were audio recorded for subsequent transcription and coding purposes. The administration of all tasks took approximately an hour.

## Results

In order to determine to what extent request and apology strategies of HL-English speakers differ and/or resemble those of dominant speakers in HL-English and in SL-Hebrew, we fitted mixed-effects logistic regression models (Baayen et al., 2008) with lme4 package (Bates et al., 2015) since the responses were mainly coded in a binary manner (1 = present, 0 = absent), i.e., syntactic structure, the use of modals, the use of *‘please’*/*‘bevakasha’*, the use of IFIDs, and the use of adverbial intensifiers. We tested the contribution of Language (English, Hebrew) together with Group (ENG-D, HEB-D, HS); these variables and their interactions were entered as fixed effects. To account for the variability within participants and scenarios, the models included crossed random intercepts for Participant and Scenario. Fitted models were compared in terms of Akaike Information Criterion (AIC) and Bayes Information Criterion (BIC), with reduced AIC and BIC values indicating a better model fit. This was supplemented by Likelihood ratio tests conducted to determine if the inclusion of a predictor significantly improved the model fit. First, we examined whether the inclusion of the random effects was permitted. This was done by comparing a baseline generalized linear model without the random intercepts (null model) with a baseline mixed-effects model that only included the random intercepts. Next, we implemented a step-wise-step-up procedure for building the mixed-effects model. The significance level of the main fixed effects was obtained using the ANOVA function. We obtained the estimated marginal means (EMM) for all pairwise comparisons using Tukey’s HSD adjustment for multiple comparisons. In the results subsection, we report the final models which were found to provide the best and most parsimonious fit for the data. We fitted linear models for the analysis of the number of propositions in apologies in English and in Hebrew, as the data were coded in a non-binary manner (0–4). We also ran models with ‘Social Status’ and ‘Social Distance’ as fixed variables, yet the inclusion of these effects and their interactions with Group did not improve the fit of the models. In the Limitations subsection, we outline possible reasons for that.

### Requests

#### Alerters, head acts, and supportive moves

Table 5 presents the descriptive statistics for the use of alerters, head acts, and supportive moves coded as 1 = response with an alerter/supportive move and 0 = response without an alerter/supportive move, and 1 = response with more than one head act and 0 = response with one head act.

**Table 5:** Alerters, head acts, and supportive moves per group per language [Mean (SD)].

	English			Hebrew		
	HS	HEB-D	ENG-D	HS	HEB-D	ENG-D
Alerters	0.71 (0.45)	0.56 (0.49)	0.43 (0.49)	0.71 (0.45)	0.58 (0.49)	0.38 (0.48)
Number of head acts	1.35 (0.47)	1.07 (0.25)	1.03 (0.19)	1.39 (0.48)	1.07 (0.26)	1.03 (0.19)
Supportive moves	0.75 (0.43)	0.54 (0.49)	0.70 (0.45)	0.72 (0.44)	0.69 (0.46)	0.60 (0.49)

Table 6 presents the final model for the use of alerters and head acts. For the use of alerters, the results showed that there was an effect of Group, yet no effect of Language and no Group*Language interaction. Follow-up pairwise Group contrasts for the use of alerters showed that there were significant differences between ENG-D and HS (*β* = −2.59; *SE* = 0.743; *Z* = –3.483, *p* = 0.0014), yet, there were no significant differences between ENG-D and HEB-D (*β* = −1.42; *SE* = 0.733; *Z* = –1.937, *p* = 0.1282) and between HEB-D and HS (*β =* −1.17; *SE* = 0.737; *Z* = –1.581, *p* = 0.2538).

**Table 6:** Final models for the use of alerters and head acts.

	Alerters				Head acts			
	**Fixed effects**							
	**Estimate**	**Std. error**	***z* Value**	**Pr(>|z|)**	**Estimate**	**Std. error**	***z* Value**	**Pr(>|z|)**
Intercept	−0.8217	0.5732	−1.434	0.151706	−3.6635	0.3373	−10.861	< 2e-16***
Group (ENG-D vs.HEB-D)	1.4199	0.7329	1.937	0.052687	0.6959	0.4137	1.682	0.0925
Group (ENG-D vs. HS)	2.5860	0.7425	3.483	0.000496***	3.0097	0.3933	7.653	1.97e-14***
	**Random effects:** Number of observations: 2160, Participant: 60; Scenario: 18							
	**Variance**	**Std. Dev**			**Variance**	**Std. Dev**		
Participant (Intercept)	5.064	2.250			0.9365	0.9678		
Scenario (Intercept)	1.047	1.023			0.2098	0.4580		

Significance codes: 0 ‘***’ 0.001.

The results for the use of head acts indicated an effect of Group, yet no effect of Language and no Group*Language interaction. Follow-up pairwise Group contrasts for the use of head acts showed that there were significant differences between ENG-D and HS (*β =* −3.010; *SE* = 0.393; *Z* = –7.653, *p* < 0.0001), and between HEB-D and HS (*β =* −2.314; *SE* = 0.366; *Z* = –6.320, *p* < 0.0001), yet, there were no significant differences between ENG-D and HEB-D (*β* = −0.696; *SE* = 0.414; *Z* = –1.682, *p* = 0.2120).

No Group and Language differences, and no Group*Language interaction were found in regard to supportive moves.

#### Syntactic structure choice of the head acts

**Figure 1:**
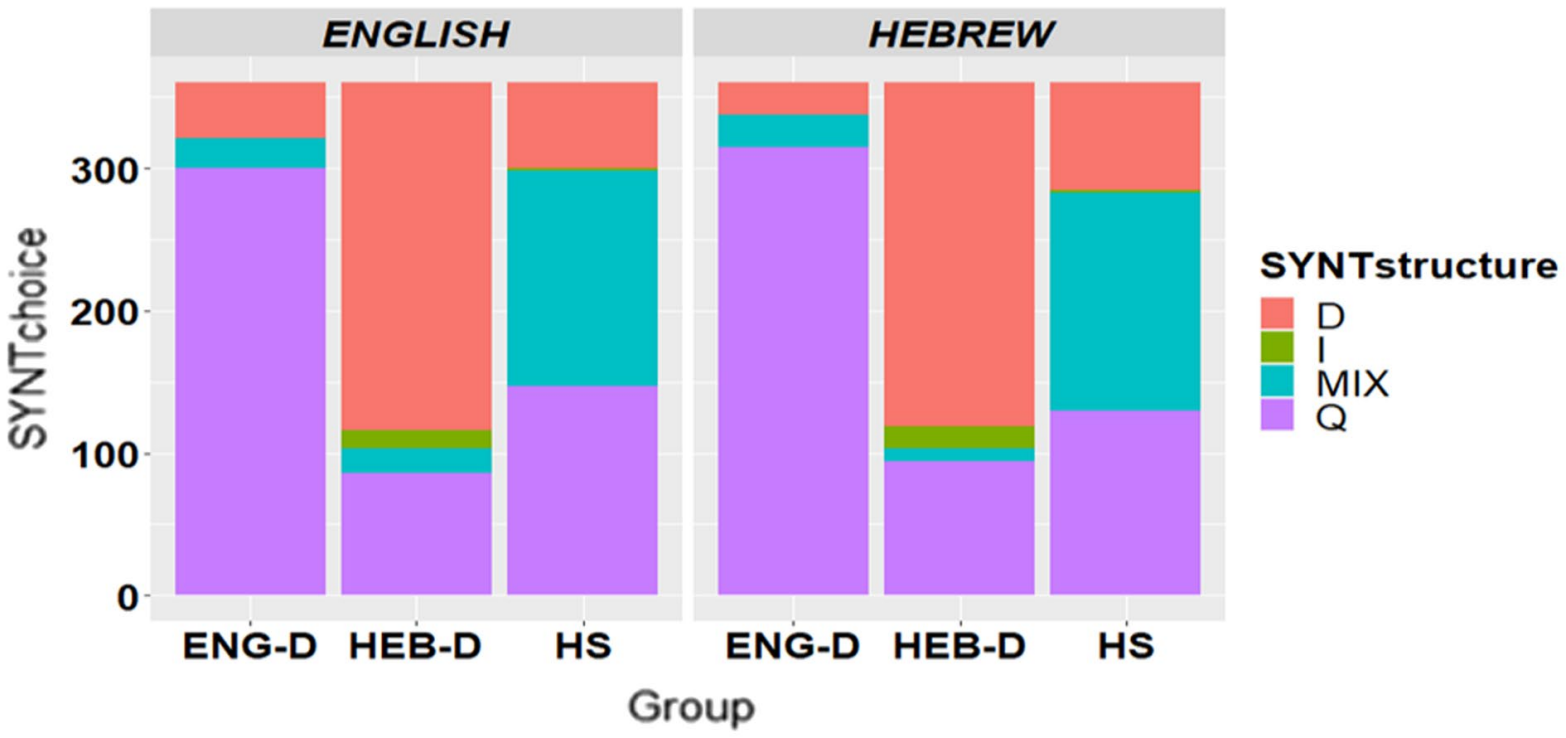
The choice of syntactic structure in request formation per group per language. D = declarative, I = imperative, Q = question (interrogative), MIX = combination of two sentence types.

To address the choice of a syntactic structure in requests, we coded participants’ head acts as declarative, interrogative, imperative, or mixed (if there were two head acts with different syntactic structures in one request scenario). Figure 1 presents the syntactic structure of the head acts used across the three groups in English and in Hebrew.

Table 7 presents the final models for the choice of the specific syntactic structure of head acts separately for interrogative, declarative, and mixed structures respectively, coded as 1 = present and 0 = absent. It is important to note that the usage of imperatives in all groups in both languages was virtually nonexistent and therefore responses in imperative forms were not analyzed statistically. Out of 2,160 scenarios, 15 instances of imperatives were found in the English data (13 in the HEB-D group and two in the HS group), and 18 instances of imperatives were documented in the Hebrew data (16 in the HEB-D group and two in the HS group). The ENG-D participants did not use imperatives at all in either of the languages.

**Table 7:** Final models for the syntactic structure choice.

	Interrogatives				Declaratives				Mixed			
	**Fixed effects**											
	**Estimate**	**Std. error**	***z* Value**	**Pr(>|z|)**	**Estimate**	**Std. error**	***z* Value**	**Pr(>|z|)**	**Estimate**	**Std. error**	***z* Value**	**Pr(>|z|)**
Intercept	2.1842	0.2998	7.286	.18e-13***	−2.6872	0.3464	−7.758	8.66e-15***	−3.2622	0.3283	−9.937	< 2e-16***
Group (ENG-D vs. HEB-D)	−3.5543	0.2981	−11.924	< 2e-16***	3.6571	0.3466	10.550	< 2e-16***	0.5781	0.4329	−1.336	0.182
Group (ENG-D vs. HS)	−2.8120	0.2935	−9.580	< 2e-16***	0.6642	0.3494	1.901	0.05727	2.8697	0.3826	7.500	6.36e-14***
Language					−0.6789	0.2904	−2.338	0.01941*				
Group HEB-D: Language Hebrew					0.6323	0.3393	1.864	0.06237				
Group HS: Language Hebrew					1.0214	0.3596	2.840	0.00451**				
	**Random Effects:** Number of observations: 2160, Participant: 60; Scenario: 18											
	**Variance**	**Std. Dev**			**Variance**	**Std. Dev**			**Variance**	**Std. Dev**		
Participant (Intercept)	0.6078	0.7796			0.6026	0.7763			0.9711	0.9855		
Scenario (Intercept)	0.7833	0.8850			0.8849	0.9407			0.2898	0.5383		

Significance codes: 0 ‘***’ 0.001 ‘**’ 0.01 ‘*’ 0.05.

For the use of interrogatives, the results showed that there was an effect of Group, yet no effect of Language and no Group*Language interaction. Follow-up pairwise Group contrasts for the use of interrogatives showed that there were significant differences between ENG-D and HEB-D (*β* = 3.554; *SE* = 0.298; *Z* = 11.924, *p* < 0.0001), between ENG-D and HS (*β* = 2.812; *SE* = 0.294; *Z* = 9.580, *p* < 0.0001), and between HEB-D and HS (*β =* 0.742; *SE* = 0.279; *Z* = 2.662, *p* = 0.0212).

The results for the use of declaratives indicated a significant Group*Language interaction, therefore pairwise Group comparisons within each language were conducted. The source of the interaction came from the HS group which paired up with the ENG-D group in English (*β* = 0.664; *SE =* 0.349; *Z* = –1.901, *p =* 0.1383), yet differed from the HEB-D group in Hebrew (*β* = 2.604; *SE =* 0.317; *Z =* 8.220, *p* < 0.0001). Similar to the use of interrogative, the two dominant groups differed from each other in both languages in the choice of declaratives.

For the choice of mixed strategy, the HS group differed from both dominant groups (HS vs. ENG-D: *β* = 2.870; *SE =* 0.383; *Z =* 7.500, *p* < 0.0001; HS vs. HEB-D: *β* = −3.448; *SE =* 0.409; *Z =* 8.423, *p* < 0.0001), while the two dominant groups were similarly unlikely to choose a mixed strategy as a request formation option (*β* = 0.578; *SE =* 0.409; *Z =* –8.423, *p =* 0.3754).

Thus, the results indicated differences between the two dominant groups: the most common syntactic structure for making requests among ENG-D was the interrogative structure while for HEB-D it was the declarative. Both dominant groups transferred this strategy from their dominant language into their weaker language. Interestingly, HS usage of both interrogatives and declaratives was between the two dominant groups in both English and Hebrew. Moreover, the pattern of mixed syntactic structure was the most preferred among HS and more common compared to the other two groups in both languages. In other words, HS diverged from both dominant groups in their overuse of mixed structure in both the HL-English and the SL-Hebrew. While ENG-D speakers preferred the interrogative structure and HEB-D preferred the declarative, HS relied on a mixed strategy that contained the interrogative and the declarative structures in one scenario (i.e., two head acts in one request).

#### The use of modals

**Figure 2:**
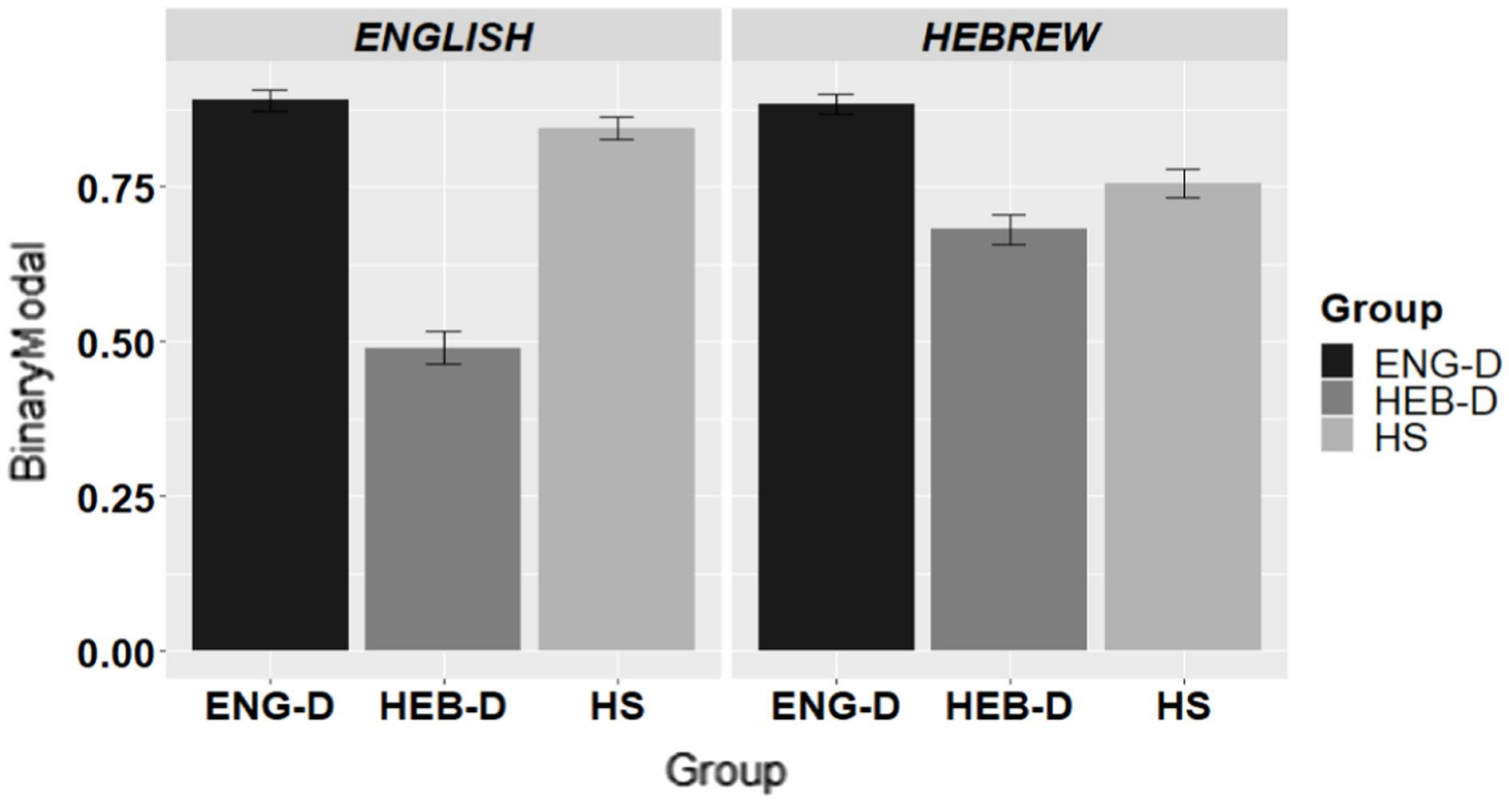
The use of modals in request formation per group per language.

Figure 2 presents the use of modals across the three groups in English and in Hebrew.

Table 8 presents the final model for the use of modals on a binary scale, coded as 1 = response with a modal and 0 = response without a modal. The results indicated no effect of Language, yet an effect of Group and a significant Group*Language interaction. Therefore, pairwise Group comparisons within each language were conducted. The results showed that ENG-D usage of modals was significantly higher than HEB-D usage of modals in English (*β* = 2.385; *SE =* 0.292; *Z* = 8.176, *p* < 0.0001) and in Hebrew (*β* = 1.453; *SE =* 0.291; *Z* = 4.987, *p* < 0.0001). However, the interaction came from the HS group which behaved differently in each language. The HS group paired up with the ENG-D group in English (*β* = 0.436; *SE =* 0.308; *Z* = 1.417, *p =* 0.3322) and with the HEB-D in Hebrew (*β* = −0.451; *SE =* 0.267; *Z* = –1.691, *p =* 0.2087).

**Table 8:** Final models for the use of modals and ‘*please’/‘bevakasha*’.

	The use of modals				The use of *‘please’*/*‘bevakasha’*			
	**Fixed effects**							
	**Estimate**	**Std. error**	***z* Value**	**Pr(>|z|)**	**Estimate**	**Std. error**	***z* Value**	**Pr(>|z|)**
Intercept	2.33706	0.25168	9.286	< 2e-16***	−1.9945	0.3155	−6.321	2.59e-10***
Group (ENG-D vs.HEB-D)	−2.38490	0.29169	−8.176	2.93e-16***	4.3432	0.4048	10.730	< 2e-16***
Group (ENG-D vs. HS)	−0.43609	0.30780	−1.417	0.15654	0.9822	0.3778	2.600	0.00932**
Language Hebrew	−0.05917	0.23893	−0.248	0.80442	−0.8082	0.2459	−3.287	0.00101**
Group HEB-D: Language Hebrew	0.93164	0.28840	3.230	0.00124**	−0.1087	0.3316	−0.328	0.74317
Group HS: Language Hebrew	−0.56647	0.31061	−1.824	0.06819	0.6967	0.3030	2.300	0.02147*
	**Random effects:** Number of observations: 2160, Participant: 60; Scenario: 18							
	**Variance**	**Std. Dev**			**Variance**	**Std. Dev**		
Participant (Intercept)	0.4006	0.6330			1.0033	1.0017		
Scenario (Intercept)	0.1881	0.4338			0.4038	0.6354		

Significance codes: 0 ‘***’ 0.001 ‘**’ 0.01 ‘*’ 0.05.

To sum up, the two dominant groups were significantly different in their usage of modals, i.e., ENG-D usage of modals was significantly higher than that of HEB-D speakers. However, contrary to both dominant groups who mirrored their strategy to both their languages, the HS were parallel to the ENG-D when speaking English and to the HEB-D when speaking Hebrew.

#### The use of ‘*please*’/‘*bevakasha*’

**Figure 3:**
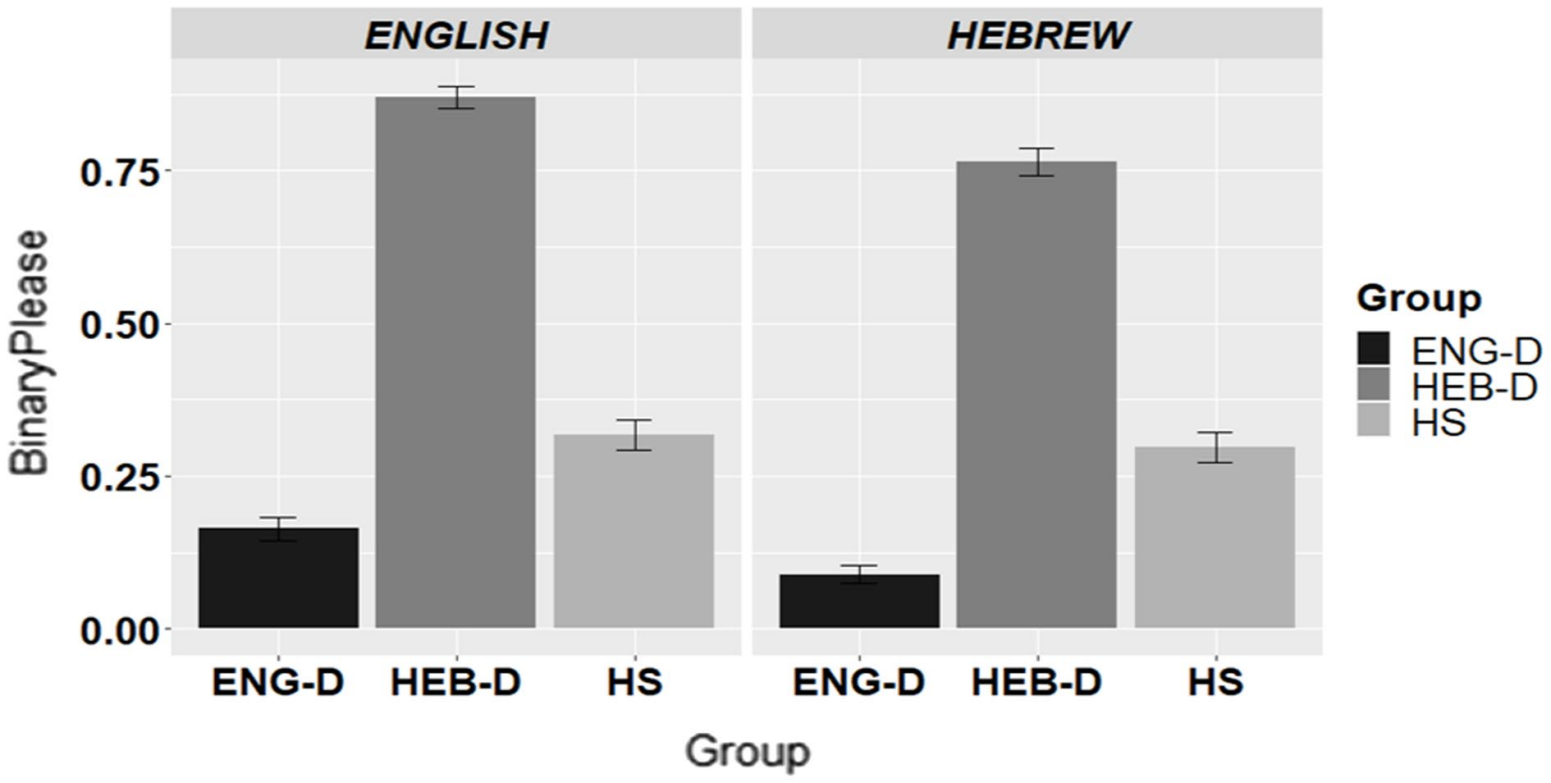
The use of ‘*please’/‘bevakasha*’ in request formation per group per language.

Figure 3 presents the use of ‘*please*’/‘*bevakasha*’ across the three groups in English and in Hebrew.

Table 8 presents the final model for the use of ‘*please*’/‘*bevakasha*’ coded as 1 = response with *‘please’*/*‘bevakasha’* and 0 = response without *‘please’*/*‘bevakasha’*. The results indicated no effect of Language, yet an effect of Group and a Group*Language interaction. The follow-up analyses showed that HEB-D usage of *‘please’*/*‘bevakasha’* was significantly higher than that of ENG-D in both English (*β* = −4.343; *SE =* 0.405; *Z* = –10.730, *p* < 0.0001) and Hebrew (*β* = −4.234; *SE =* 0.408; *Z* = –10.384, *p* < 0.0001). However, the HS group differed from both dominant groups in both languages by being somewhere in between, i.e., HS usage of *‘please’*/*‘bevakasha’* was higher than ENG-D (*β* = −0.982; *SE =* 0.378; *Z* = –2.600, *p* = 0.0253) and lower than HEB-D (*β* = −3.361; *SE =* 0.391; *Z* = –8.595, *p* < 0.0001) in English, as well as higher than ENG-D (*β* = −1.679; *SE =* 0.399; *Z* = –4.206, *p* = 0.0001) and lower than HEB-D (*β* = 2.556; *SE =* 0.374; *Z* = 6.840, *p* < 0.0001) in Hebrew.

To sum up, the findings for the usage of *‘please’*/*‘bevakasha’* showed that HEB-D resorted to the use of *‘please’*/*‘bevakasha’* significantly more than ENG-D in forming requests, while the HS were in between the two dominant groups in both languages.

### Apologies

#### The use of propositions

**Figure 4:**
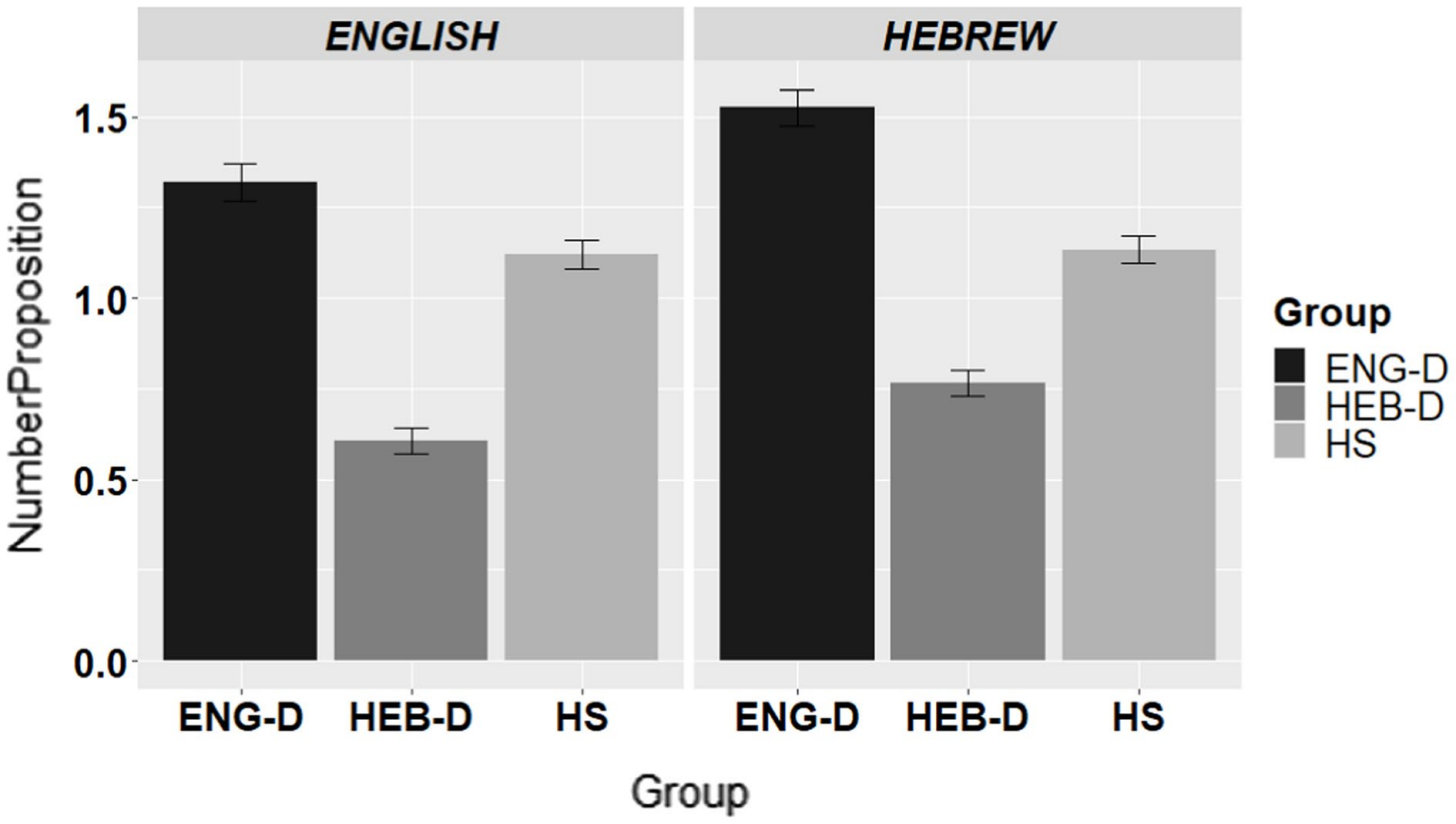
The use of propositions in apology formation per group per language.

To address the issue of the use of propositions, we coded the number of propositions used in each apology from 0 to 4 (i.e., offering explanation, taking responsibility, offering repair or compensation, and promising forbearance) giving 1 point for each proposition. Figure 4 presents the number of propositions across the three groups in English and in Hebrew.

The results in Figure 4 show that while the ENG-D group had the highest frequency of propositions, the HEB-D group had the lowest. This trend was observed in both languages. The HS group was in between these two dominant groups in both English and Hebrew. This was confirmed by the statistical analysis in which we fitted linear regression models, as the data were coded in a non-binary manner (0–4), with Group, Language, and the interaction between them as fixed variables. Table 9 presents the final model for the number of propositions used, coded as 0 = response without a proposition, 1 = response with one proposition, 2 = response with two propositions, 3 = response with three propositions, and 4 = response with four propositions. The results showed an effect of Group, an effect of Language, and a significant Language*Group interaction. Follow-up pair-wise contrasts indicated that the groups differed from each other in both languages (all comparisons at *p* < 0.001).

**Table 9:** Estimate parameters for the use of propositions.

	Est.	25%	75%	*t* value	*p*
Intercept	1.32	1.29	1.35	31.96	0.00
Group (ENG-D vs.HEB-D)	−0.71	−0.75	−0.67	−12.23	0.00
Group (ENG-D vs. HS)	−0.20	−0.24	−0.16	−3.43	0.00
Language	0.21	0.17	0.24	3.52	0.00
Group HEB-D: Language	−0.04	−0.10	0.01	−0.54	0.59
Group HS: Language	−0.19	−0.25	−0.14	−2.32	0.02

To sum up, the findings for the usage of propositions showed that the ENG-D group adhered to the use of propositions significantly more than the HEB-D group in forming apologies, while the HS were in between the two dominant groups in both languages.

#### The use of IFIDs

**Figure 5:**
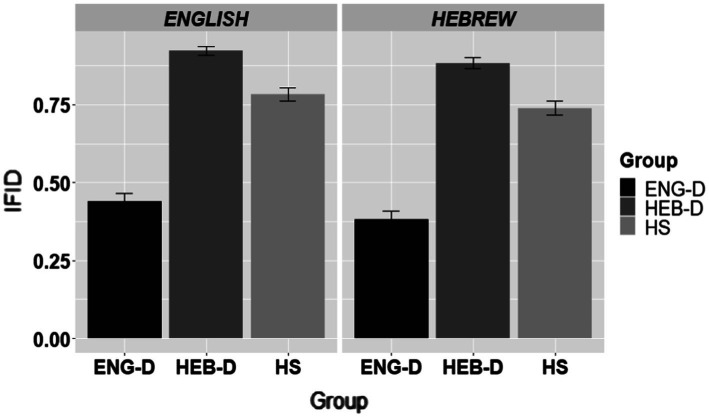
The use of illocutionary force-indicating devices (IFIDs) in apology formation per group per language.

Figure 5 presents the use of IFIDs across the three groups in English and in Hebrew.

The results in Figure 5 show that IFIDs were frequently used by HEB-D speakers while less so by ENG-D speakers in both languages. The HS were in between these two dominant groups in both English and Hebrew. This was confirmed by the statistical analysis shown in Table 10 which presents the final model of the use of IFIDs, coded as 1 = response with an IFID and 0 = response without an IFID. The results indicated an effect of Group, yet no effect of Language and no significant Group*Language interaction. The results showed that the HEB-D’s usage of IFIDs was significantly higher than that of ENG-D (*β* = −3.48; *SE =* 0.408; *Z* = −8.535, *p* < 0.0001). The HS group differed from both dominant groups in both languages by being somewhere in between, i.e., HS usage of IFIDs was higher than ENG-D (*β* = −2.13; *SE =* 0.390; *Z* = −5.475, *p* < 0.0001) and lower than HEB-D (*β* = 1.35; *SE =* 0.406; *Z* = 3.317, *p* = 0.0026).

**Table 10:** Final models for the use of IFIDs and adverbial intensifiers.

	The use of IFIDs				The use of adverbial intensifiers			
	**Fixed Effects**							
	**Estimate**	**Std. error**	***z* Value**	**Pr(>|z|)**	**Estimate**	**Std. error**	***z* Value**	**Pr(>|z|)**
Intercept	−0.2944	0.3713	−0.793	0.42785	−0.7055	0.3290	−2.144	0.0320*
Group (ENG-D vs.HEB-D)	3.4799	0.4077	8.535	< 2e-16***	1.7312	0.3720	4.653	3.27e-06***
Group (ENG-D vs. HS)	2.1332	0.3896	5.475	4.38e-08***	1.5971	0.3694	4.323	1.54e-05***
Language HEBREW	−0.3737	0.1211	−3.086	0.00203**	−0.8207	0.1929	−4.255	2.09e-05***
Group HEB-D: Language HEBREW					0.8548	0.2659	3.215	0.0013**
Group HS: Language HEBREW					−0.2279	0.2588	−0.881	0.3786
	**Random effects:** Number of observations: 2160, Participant: 60; Scenario: 18							
	**Variance**	**Std. Dev**			**Variance**	**Std. Dev**		
Participant (Intercept)	1.29	1.136			1.0337	1.017		
Scenario (Intercept)	1.10	1.049			0.7175	0.847		

Significance codes: 0 ‘***’ 0.001 ‘**’ 0.01 ‘*’ 0.05.

To sum up, the findings of the usage of IFIDs showed that HEB-D adhered to the use of IFIDs significantly more than ENG-D, while the HS were in between the two dominant groups in both languages.

#### The use of adverbial intensifiers

**Figure 6:**
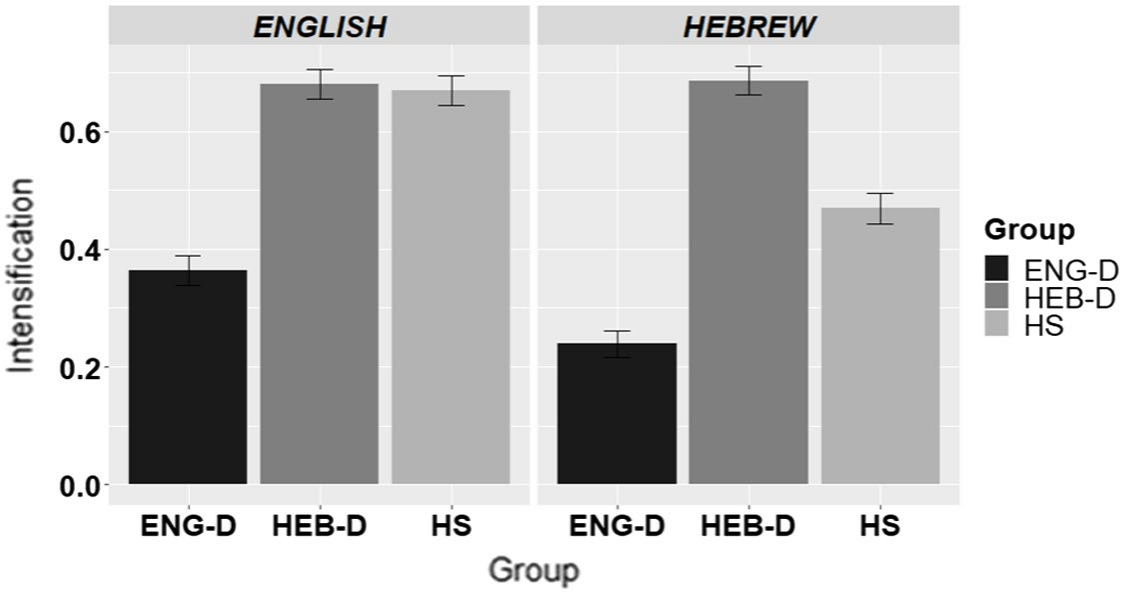
The use of adverbial intensifiers in apology formation per group per Language.

Figure 6 presents the use of adverbial intensifiers across the three groups in English and in Hebrew.

To investigate the usage of intensifiers, we also used a binary coding scheme (1 = if an adverbial intensifier was present, and 0 = if it was absent in an apology response). The results in Figure 6 show that the use of adverbial intensifiers was the highest in the HEB-D group, while it was the lowest in the ENG-D group in both languages. As for the participants in the HS group, their usage of adverbial intensifiers was between these two dominant groups in Hebrew, yet on par with the HEB-D participants in English. This was confirmed by the statistical analysis shown in Table 10. The results indicated no effect of Language, yet an effect of Group and a significant Group*Language interaction. Therefore, pairwise Group contrasts within each language were conducted. The results showed that the HEB-D usage of adverbial intensifiers was significantly higher than that of ENG-D in English (*β* = −1.731; *SE =* 0.372; *Z* = –4.653, *p* < 0.0001) and in Hebrew (*β* = −2.586; *SE =* 0.380; *Z* = –6.802, *p* < 0.0001). The source of the Group*Language interaction came from the HS group which paired up with the HEB-D in English (*β* = 0.134; *SE =* 0.370; *Z* = 0.363, *p =* 0.9301) and differed from both dominant groups in Hebrew by being somewhere in between (both comparisons were *p* < 0.001).

To sum up, the findings of the usage of adverbial intensifiers showed that HEB-D adhered to the use of adverbial intensifiers significantly more than ENG-D. The HS paired up with the HEB-D group in English, while they were in between the two dominant groups in Hebrew.

Table 11 presents examples of prototypical requests and apologies produced by ENG-D, HEB-D, and HS. A prototypical request produced by an ENG-D participant had an interrogative form and included a modal, yet no *‘please’*/*‘bevakasha’*, while a prototypical request produced by a HEB-D participant was formulated as a declarative and included *‘please’*/*‘bevakasha’*, yet no modals. A prototypical HS request included a mixed strategy containing both the declarative and the interrogative structures, and an ‘in-between’ *‘please’*/*‘bevakasha’* strategy in both HL-English and SL-Hebrew. HS strategy with regard to modals paired up with the ENG-D in English and with the HEB-D in Hebrew.

**Table 11:** Examples of prototypical requests and apologies per group per language (Scenario 2).

		Requests
ENG-D	English (P53)	May I use your computer just for a few minutes?
	Hebrew (P6)	?אני יכולה להשתמש במחשב שלך לדקה, שתי דקות Ani yexola lehishtamesh bamaxshev shelxa ledaka, shtei dakot? *Can I use your computer for a minute, two minutes?*
HEB-D	English (P24)	I need to use your computer for a sec please.
	Hebrew (P16)	.אני יודעת שממש חשוב לך להשתמש במחשב, אבל אשמח לאפשרות לקבל אותו לכמה דקות בבקשה Ani yoda’at shemamash xashuv lexa lehishtamesh bamaxshev, aval esmax le’efsharut lekabel oto lekama dakot bevakasha. *I know that it is extremely important for you to use the computer, but I’ll be happy to have the opportunity to get it for a few minutes please*.
HS	English (P43)	Hi honey. I need your computer. Please, it’s important. Can I have it? Just for a few minutes.
	Hebrew (P33)	?היי חומד. המחשב שלי לא נדלק אז אני אשמח לקחת את שלך לרגע. יש מצב שאתה נותן לי אותו לכמה דקות Hi xomed. Hamaxshev sheli lo nidlak az ani esmax lakaxat et shelxa lerega. Yesh matzav she’ata noten li oto lekama dakot? *Hi hon. My computer is not turning on so I’ll be happy to take yours for a second. Is there a chance you give it to me for a few minutes?*
		**Apologies**
ENG-D	English (P59)	Oh my goodness. I do not know how it happened. I’ll do another essay and write your name on it.
	Hebrew (P38)	?אופס, זה לא היה בכוונה. אולי אני יכולה לעזור לך לכתוב את זה עוד פעם Oops, ze lo haya bexavana. Ula’i ani yexola la’azor lexa lixtov et ze od pa’am? *Oops, it wasn’t on purpose. Maybe I can help you rewrite it again?*
HEB-D	English (P15)	Ohh, I’m very very sorry for deleting your essay. I do not have any words to beg your pardon.
	Hebrew (P15)	.וואו, אני מאוד מצטער על מחיקת החיבור. אני מבקש את סליחתך Wow, ani me’od mitzta’er al mexikat haxibur. Ani mevakesh et slixatxa. *Wow, I’m so sorry about the essay deletion. I request your forgiveness.*
HS	English (P1)	Listen, I accidentally deleted your file. It wasn’t on purpose. I’m really sorry.
	Hebrew (P50)	.בטעות נמחק לי החיבור שלך. אני ממש מצטער. אם אתה רוצה שאני אעזור לך לשחזר אני איתך Beta’ut nimxak li haxibur shelxa. im ata rotze she’ani e’ezor lexa leshaxzer ani itxa. *By mistake your essay has been deleted to me. I’m really sorry. If you want me to help you rconstruct it I’m with you.*

A prototypical apology produced by an ENG-D participant included propositions, yet no IFIDs and no adverbial intensifiers, while a prototypical apology produced by a HEB-D participant included IFID/IFIDs and adverbial intensification, yet no propositions. A prototypical HS apology reflected a mixed strategy, i.e., it included both IFIDs and propositions, and an ‘in-between’ adverbial intensification strategy in Hebrew, while on par with HEB-D in English.

## Discussion

The overall aim of this study was to investigate HL-English speakers’ pragmatics *via* request and apology realization patterns in both of their languages, i.e., English and Hebrew, as compared to two dominant groups: Hebrew-dominant speakers (HEB-D) and English-dominant speakers (ENG-D). The results indicated that dominant speakers of Hebrew and English use different strategies in request and apology formation. Furthermore, the results showed that dominant speakers transfer strategies from their dominant language into their weaker one. As for the HL-English speakers (HS), the results showed a complex picture. In some cases, they paired with dominant speakers, yet sometimes they favored a different strategy. These novel strategies suggest that HL pragmatics is a hybrid system which embodies a mixture of the HL and the SL pointing at a bi-directional cross-linguistic transfer. This hybridity enables HL speakers to draw on pragmatic patterns from their two languages in order to accommodate both languages. Our study shows that the nature of HL pragmatics can be studied when considering the pragmatic competence in both languages of HL speakers.

### Requests strategies

For the analysis of request strategies, we compared the choice of a syntactic structure of head acts, the use of modals, and the use of *‘please’*/*‘bevakasha’* across the three groups in both of their languages. These measures of analyses were chosen as they were hypothesized to reflect differences in request formation in English and in Hebrew (see the studies reviewed in the Introduction subsection).

Starting with the choice of the syntactic structure, differences were found between the two dominant groups (ENG-D and HEB-D) reflecting differences in the cultural perception of appropriateness of English-dominant and Hebrew-dominant speakers. However, before exploring the preferred syntactic structure in each of the languages, it is important to mention that even though requests in both English and Hebrew can be grammatically realized with imperatives, interrogatives, and declaratives (Blum-Kulka and Olshtain, 1984; Curl and Drew, 2008), imperatives were hardly ever used by Hebrew-dominant speakers, even less so by HL-English speakers, and not even once by English-dominant speakers. In a continuation to directness ideas, a request in the form of an imperative is the most direct and explicit, and therefore is considered to be the least polite (Blum-Kulka and Olshtain, 1984). This indicates that usage of imperative structure requires much effort to modify, and therefore tends to be avoided. Blum-Kulka et al. (1989) and Leech (1980) noted that since interrogatives are most often requests for permission they increase the degree of optionality, and therefore are perceived as being more polite and indirect than declaratives. As expected from the literature review summarized in the Introduction, and as can be seen from the results of the current study, the preferred structure for requests among English-dominant speakers was the interrogative, while for the Hebrew-dominant speakers it was the declarative. The HL-English speakers had at their disposal the strategies of two languages; however, they favored a mixed strategy containing the interrogative and the declarative structures in the same request. This hybrid strategy allowed the HL-English speakers to transfer the same strategy between both their languages.

Differences in the usage of modals were also found between the two dominant groups. English-dominant speakers used modals significantly more frequently than Hebrew-dominant speakers in their dominant language and in their weaker one. This is consistent with Turnbull and Saxton’s (1997) proposal that English speakers use expressions of modality to do ‘facework’ since they convey the notion of permission, ability, probability, possibility, etc., and therefore further emphasize the indirectness of the utterance. The HL-English speakers’ usage of modals was found to be on par with the English-dominant speakers in English and with the Hebrew-dominant speakers in Hebrew. Contrary to the trend shown for the choice of the syntactic structure, in the usage of modals, the HS group did not develop a unique and hybrid strategy, but rather adopted the customary behavior of each language.

Differences in the usage of *‘please’*/*‘bevakasha’*, as predicted, were also found between the two dominant groups: Hebrew-dominant speakers resorted to the use of *‘please’*/*‘bevakasha’* significantly more frequently than English-dominant speakers. This matches House’s (1989) idea that the politeness marker ‘*please*’ is most appropriate in mitigating situations where the function of the request is clear, and less so with interrogatives since it might reveal the true nature of the request. The HL-English speakers, on the other hand, were found to be in between these two dominant groups in both languages.

The results for request formation showed that HL-English speakers developed a unique and hybrid intercultural linguistic style reflecting strategies of both languages (their HL-English and their SL-Hebrew): In both languages, HL-English speakers adhered to a mixed strategy containing the interrogative and the declarative structures in the same request, and their usage of ‘*please’*/*‘bevakasha’* was in between the two dominant groups. This is in line with Taguchi and Roever’s (2017) suggestion that HL pragmatics is a hybrid system reflecting norms of both HL and SL that develops in blended social contexts (i.e., social interactions with both languages’ communities) and is mediated by bi-directional cross-linguistic influence. Furthermore, the results were in line with Pinto and Raschio’s (2007) findings showing that when HL-Spanish speakers came into contact with English both pragmatic systems were affected. However, we also see that in some cases HL speakers adopt the customary behavior of each language, as it is the case for the use of modals.

### Apology strategies

For the analysis of apology strategies, we compared usage of apology expressions, number of propositions added (i.e., offering explanation, taking responsibility, offering repair or compensation, and promising forbearance), and usage of adverbial intensifiers across the three groups in their both languages. These measures of analyses were chosen as they were hypothesized to reflect differences in apology formation in English and in Hebrew (see the studies reviewed in the Introduction subsection).

In order to interpret the results of the usage of propositions and the usage of IFIDs, it is important to consider them together, since, as we shall see, the results of the HL-English speakers’ usage of both are connected. In line with previous studies, there were significant differences between the two dominant groups (ENG-D and HEB-D) with respect to both usage of propositions and usage of IFIDs. As Blum-Kulka and Olshtain (1984) noted there are two options for apologizing: the first one is direct and explicit and involves the use of an IFID, while the second one is indirect and involves ‘going around’ by offering one or more of four propositions. However, a speaker might also choose to incorporate both strategies within one apology. Since Hebrew is reported to be more direct and straightforward than English (Mills and Grainger, 2016), it was not surprising to find that Hebrew-dominant speakers preferred to apply the direct strategy in the form of an IFID, while English-dominant speakers tended to apply the indirect strategy in the form of propositions to their apologies. In fact, this trend was so salient that it looked as if the Hebrew-dominant speakers believed that an apology must comprise an IFID as a compulsory component, optionally followed by the other strategy, while the English-dominant speakers believed that propositions such as explaining, taking responsibility or offering repair were more appropriate than IFIDs. The HL-English speakers, however, did not replicate either one of the dominant groups’ strategies. Instead, they seemed to develop their own strategy of apologies by combining both IFIDs and propositions. This hybridity was applied by HS in both their languages. In other words, the HL-English speakers were found to be in between the two dominant groups in the use of both IFIDs and propositions.

Differences in the usage of intensifiers, again as expected, were found between the two dominant groups; Hebrew-dominant speakers favored the use of intensifiers significantly more than English-dominant speakers. However, it is important to note here that this study focused on adverbial intensifiers expressions only (lexical, and not phrasal), such as ‘so’, ‘very’, ‘really’, ‘terribly’, ‘extremely’, ‘totally’, ‘deeply’, ‘highly’ etc., and disregarded other intensifying expressions. Since English is less direct than Hebrew, and English native speakers’ usage of IFIDs is reduced as compared to native Hebrew speakers, it is logical to assume that native English speakers might choose to incorporate less direct intensifying strategies in their apologies. For example, they might choose expressions that convey concern for the hearer, which are external to the IFID or the other strategies used such as ‘Have you been waiting long?’. However, this trend was not checked in the current research. The usage of adverbial intensifiers among the HS group was between the two dominant groups in Hebrew, yet, on par with the Hebrew-dominant speakers in English. Future studies should expand the research on intensification by looking into the usage of intensifiers in all their forms.

Thus, the picture for apology formation in HL-English speakers was similar to that of request realization. In some aspects, HL-English speakers adhered to the strategy of dominant speakers of the languages, as is the case for the use of adverbial intensifiers in English, yet in other cases their realization patterns of apologies reflected a blended pragmatic system which suited both languages, as is the case of IFIDs and propositions.

### Heritage language pragmatics: Economy principle/dual identity/intercultural style hypothesis

The results indicated that the dominant groups had different strategies for making requests and apologies which they systematically transferred from their dominant language into their weaker one, confirming the cross-cultural and cross-linguistic differences between request and apology strategies in English and in Hebrew. As for HL-English speakers, new blended conventions of request and apology were detected.

The HL-English speakers’ pragmatic hybridity might be explained in the light of the ‘cognitive economy principle’. The HL speakers’ proficiency in two languages enables them to combine their knowledge into one strategy and use it for both languages. The principle of economy has been proposed to influence the restructuring of HL grammars. We speculate that the driving source of the hybrid nature of pragmatics in HL-Speakers in their HL-English and SL-Hebrew might be related to the proposed economy principles. Blended new conventions, formed as a result of a bi-directional cross-linguistic transfer, might be less cognitively costly as compared to the storage and retrieval of two separate systems. However, we agree that ‘cognitive economy’ is an elusive concept (Westergaard, 2021), and therefore call for future studies to further investigate this possibility.

Alternatively, the HL-English speakers’ hybridity might also be connected to issues of dual identity as it fulfills not just linguistic but also identity needs. As Val and Vinogradova (2010) suggested, the core identity of HL speakers involves the process of constant negotiation and self-positioning within a bilingual and bicultural environment. Previous studies investigating the identity of HL speakers note their complex identities. For example, Kang (2013) showed that HL-Korean speakers residing in the USA perceived themselves as different from both Koreans and “mainstream Americans.” The identity perception of HL-English speakers residing in Israel was demonstrated for preschool children (see Altman et al., 2021). The authors showed that English-Hebrew bilingual children residing in Israel gave similar ratings to Societal/Israeli and Home/American identities, pointing to the existence of bicultural identity already in young children. It is highly plausible that the pragmatic competence of adult HL-English speakers residing in Israel in the current study reflects their multiple sociolinguistic identities. Future research should address how the sociolinguistic identity is related to the pragmatic competence of an HL speaker, i.e., whether there are differences between HL speakers who value their HL identity higher versus those who value their SL identity higher. Yet, our research cannot support or rule out this hypothesis, and future studies also need to incorporate data on the identity of HL-speakers to test whether linguistic hybridity reflects HL-speakers’ complex dual identity.

Our findings for the hybrid/blended pragmatic conventions highlight the importance of analyzing bi-directional interaction in pragmatic development and might also be related to the ‘Intercultural Style Hypothesis’. Intercultural style has been shown to develop when speakers master proficiency in two languages or more. Since bilinguals/multilinguals are exposed to different ways of achieving pragmatic competence in different languages, they could use an underlying conceptual base and develop an intercultural style which explains the similarities of their realization patterns in all their languages. Monolinguals do not need to use these strategies since their realizations correspond to their experience in one single language (Cenoz, 2008).

## Limitations and future studies

Despite the fact that the study contributes to the understanding of the existing literature on politeness and language maintenance among adult HL speakers, it is not without limitations. The results showed no effects of Social Status and Social Distance parameters which was rather surprising. One possible explanation is that the design of this study did not control for the severity (imposition for requests, and offense for apologies) and the settings of the situations. We believe that future studies should control for situational severity and situational settings in order to detect Social Status and Social Distance effects in a more rigorous manner. Furthermore, the focus of this study regarding intensifiers was limited to adverbial intensifier expressions only (lexical, and not phrasal), and disregarded other intensifying expressions such as expressions that convey concerns for the hearer which are external to the IFID or other strategies used. This might have caused a partial picture of the usage of intensifications. In future studies, it might be worthwhile to look into the usage of intensifiers in a more comprehensive way in order to get a fuller and more accurate picture. Future studies might also want to distinguish between different types of declaratives as they behave differently with respect to pragmatics. Finally, our recommendations are to expand the investigation of requests to other linguistic categories following Blum-Kulka and Olshtain (1984) such as strategy type (direct, conventionally indirect, non-conventionally indirect), point of view operation, downgraders, etc. as well as to other speech acts and/or languages.

## Conclusions

The study adds to the existing literature on politeness and language maintenance among bilingual speakers. The design of the current study, which included the investigation of both languages of three groups of bilinguals, has provided valuable insights into the pragmatics of dominant speakers, L2 learners, and HL speakers. From a theoretical perspective, the study sheds light on the pragmatic competence of HL speakers in language contact situations by examining cross-linguistic and cross-cultural differences in order to provide a greater understanding of the mechanisms responsible for shaping speech act realizations. The results indicate that dominant speakers of Hebrew and English adhere to different strategies for making requests and apologies and that they systematically transfer these strategies from their dominant language into their weaker one, confirming the cross-cultural and cross-linguistic differences between request and apology strategies in English and in Hebrew. For the HL-English speakers, the picture was more complex: in some cases, strategies of HL-English speakers paired up with dominant speakers in HL-English and/or SL-Hebrew, while in other cases HL-English speakers developed a unique and hybrid linguistic style reflecting pragmatic conventions of both their languages, HL-English and SL-Hebrew. From a pedagogical perspective, the current study contributes to the field of teaching pragmatic skills to HL speakers and L2 learners, helping educators develop research-supported curricula that facilitate appropriate politeness strategies.

We believe that the main strength of the current study lies in its methodology: testing both languages of three groups with different levels of dominance. This design enabled us to investigate the two linguistic systems simultaneously and draw conclusions about their nature. Despite the assumption that HL speakers diverge in their HL and perform on par with dominant speakers in their SL, the current study shows that subtle differences may be observed in both languages. Thus, we highlight here the importance/advantages of investigating both languages of HL speakers in future studies in order to obtain a fuller picture of this unique bilingual group.

## Data availability statement

The raw data supporting the conclusions of this article will be made available by the authors, without undue reservation.

## Ethics statement

The current study was reviewed and approved by Bar Ilan University. The participants provided their written informed consent to participate in this study.

## Author contributions

SBO and NM conceptualized the study and wrote the manuscript. SBO developed the tasks, collected and processed the data. NM was involved in data analysis. All authors contributed to the article and approved the submitted version.

## Funding

The study was partially supported by the Israel Science Foundation (ISF) No. 552/21 “Towards Understanding Heritage Language Development: The Case of Child and Adult Heritage Russian in Israel and the USA” granted to NM.

## Conflict of interest

The authors declare that the research was conducted in the absence of any commercial or financial relationships that could be construed as a potential conflict of interest.

## Publisher’s note

All claims expressed in this article are solely those of the authors and do not necessarily represent those of their affiliated organizations, or those of the publisher, the editors and the reviewers. Any product that may be evaluated in this article, or claim that may be made by its manufacturer, is not guaranteed or endorsed by the publisher.

## Figures and Tables

**Table:** 

Social status	Social distance	Gender	Speech act	The situation
S > H	L-SD	F	R	A parent asks his/her daughter to plan her birthday party on her own.
S > H	L-SD	F	A	The parent forgets about it and does not show up for the party.
S > H	L-SD	M	R	An uncle/aunt asks a young nephew to use his computer for a couple of minutes.
S > H	L-SD	M	A	The uncle/aunt deletes the essay he was writing.
S > H	M-SD	F	R	A manager asks an employee to take notes during an important meeting.
S > H	M-SD	F	A	The manager spills coffee on the employee’s dress.
S > H	M-SD	M	R	A school principal asks a teacher to come to his/her office at four o’clock to discuss a school project.
S > H	M-SD	M	A	The school principal has kept the teacher waiting for an hour because of an unexpected meeting.
S > H	H-SD	F	R	A senior manager asks a new trainee to borrow her brand-new laptop.
S > H	H-SD	F	A	The senior manager accidently smashes the laptop screen.
S > H	H-SD	M	R	A university professor asks a new student to give his presentation a week earlier than scheduled.
S > H	H-SD	M	A	The university professor realizes that the original date was the correct one.
S < H	L-SD	F	R	The speaker asks his/her mother to borrow her car.
S < H	L-SD	F	A	The speaker drives the car into a tree.
S < H	L-SD	M	R	The speaker asks his/her father to borrow his boat for a date.
S < H	L-SD	M	A	The speaker breaks the steering wheel.
S < H	M-SD	F	R	A teacher asks the school principal to leave early so he/she can go to a conference.
S < H	M-SD	F	A	The teacher finds out that the conference is scheduled for the following week.
S < H	M-SD	M	R	The speaker asks his elderly neighbor for help in statistics.
S < H	M-SD	M	A	The speaker forgets to show up on time and is an hour late.
S < H	H-SD	F	R	A new student asks a lecturer to borrow her book.
S < H	H-SD	F	A	The student forgets to return the book.
S < H	H-SD	M	R	An employee asks a new manager for a loan.
S < H	H-SD	M	A	The employee returns the money later than agreed.
S=H	L-SD	F	R	A sibling asks his/her sister to be first in the shower.
S=H	L-SD	F	A	The sibling realizes that he/she used up all the hot water.
S=H	L-SD	M	R	A friend asks another friend to make use of his house in the countryside.
S=H	L-SD	M	A	The friend spills ink on an expensive carpet.
S=H	M-SD	F	R	An employee asks a coworker from a different department to cover for him/her for an hour while going on a personal errand.
S=H	M-SD	F	A	The employee returns after 3 hours.
S=H	M-SD	M	R	The speaker asks his neighbor for help to move a bookcase out of the apartment.
S=H	M-SD	M	A	The speaker accidently closes a drawer on the neighbor’s hand.
S=H	H-SD	F	R	The speaker asks a bus passenger to swap seats with her.
S=H	H-SD	F	A	The speaker steps on the passenger’s foot.
S=H	H-SD	M	R	The speaker asks someone on the street to make a quick call from his cellphone.
S=H	H-SD	M	A	The speaker realizes that he had his/her cellphone in his/her pocket all along.

S = Speaker, H = Hearer, L-SD = Low Social Distance, M-SD = Medium Social Distance, H-SD = High Social Distance, F = Female, M = Male, R = Request, A = Apology.
